# Supplementary material for: Repurposing cancer drugs identifies kenpaullone which ameliorates pathologic pain in preclinical models via normalization of inhibitory neurotransmission
Source: Nat Commun. 2021 Oct 27;12:6208. doi: 10.1038/s41467-021-26270-3 (PMC8551327; doi:10.1038/s41467-021-26270-3)
Supplement: Supplementary file 1 — Supplementary Information [file 41467_2021_26270_MOESM1_ESM.pdf]

Title:

Repurposing cancer drugs identifies kenpaullone which ameliorates pathologic pain in preclinical models via normalization of inhibitory neurotransmission

SUPPLEMENTARY INFORMATION

Authors:

Michele Yeo[1,¶,#], Yong Chen[1,¶,#], Changyu Jiang[2], Gang Chen[2], Kaiyuan Wang[2], Sharat Chandra[2], Andrey Bortsov[2], Maria Lioudyno[3], Qian Zeng[1], Peng Wang[1], Zilong Wang[1,2], Jorge Busciglio[3], Ru-Rong Ji[2,4,¶], Wolfgang Liedtke[1,2,4,5,6,*,¶]

Affiliations:

1 Departments of Neurology, Duke University Medical Center, Durham NC; USA

2 Department of Anesthesiology (Center for Translational Pain Medicine), Duke University Medical Center, Durham NC; USA

3 Department of Neurobiology & Behavior, Institute for Memory Impairments and Neurological Disorders (iMIND), Center for the Neurobiology of Learning and Memory, University of California at Irvine, Irvine CA; USA

4 Department of Neurobiology, Duke University Medical Center, Durham NC; USA

5 Duke Neurology Clinics for Headache, Head-Pain and Trigeminal Sensory Disorders, Duke University Medical Center, Durham NC; USA

6 Duke Anesthesiology Clinics for Innovative Pain Therapy, Duke University Medical Center, Durham NC; USA

# These authors contributed equally.

¶ corresponding authors: myeo@duke.edu, yong.chen@duke.edu, ru-rong.ji@duke.edu, wolfgang.liedtke@regeneron.com

* lead contact: wolfgang.liedtke@regeneron.com

Supplementary Materials

Supplementary Data File S1

Supplementary Data File S2

Supplementary Data File S3

Supplementary Methods

Supplementary Tables

Supplementary Figures

Supplementary Methods

Screening in primary cortical neurons from *Kcc2*-LUC transgenic mice

Transgenic mice that express red-shifted luciferase (LUC) under the control of the *Kcc2* promoter (-2052/+476, described in [1]), inserted into the Rosa26 locus, were previously described by our laboratory [2,3]. We generated primary cortical neuronal cultures from newborn (p0) mice of this line following [2,3].

Cytosine arabinoside (2.5 µM) was added to cultures on the second day after seeding [2d *in vitro* (DIV)] to inhibit the proliferation of non-neuronal cells. Cell suspension was plated at a density of 1×10^6 cells/ml onto 24-well tissue-culture dishes coated with poly-D-lysine. Cortical neuronal cultures prepared by this method yielded a majority population of neuronal cells, with negligible glia contamination, as evidenced by the absence of GFAP by Western blotting.

After a week in culture, neurons were treated with compounds (100nM for 48h). LUC activity was then determined for each compound. Culture supernatant was removed and cells were lysed with 150µl lysis buffer (Targeting Systems CA, USA cat. CLR1). LUC activity was measured with a *Red*Luciferase Assay kit (Targeting Systems cat. FLAR) according to the manufacturer's instructions. A Veritas microplate luminometer was used to measure luminescence; for each treatment triplicates of 40µl cell lysates (from each 24-well tissue-culture dish) were used and 25µl substrate was injected per well. To evaluate the quality of screening methodology, Z' factor [4] was ascertained using 0.5% (v/v) DMSO as negative control and Trichostatin-A as positive control.

The primary screen encompassed three levels of LUC measurements. The first level of screening yielded a total of 137 compounds with cut-off RLU >125% LUC activity. These 137 compounds were subject to second round of screening, which was carried out in duplicate independent assays to yield the top 103 compounds sorted for highest RLU. The 103 compounds were then subjected to a third round of screening carried out in duplicate independent assays to yield the top 40 compounds which were again ranked. The best 22 of these 40 compounds (ranked for highest RLU activity) were subjected to secondary screening: Cultured neurons were treated with the 22 compounds; RT-qPCR and Clomeleon imaging methodologies [1] were used to determine effects of compounds on *Kcc2* mRNA expression and [Cl-]i, respectively. Each compound was ranked based on composite scores from primary and secondary screening.

Human neuronal cultures

The neuron-enriched cultures were established from fetal cortical specimens at 15–20 weeks of gestation. The protocols for tissue processing complied with all US federal and institutional guidelines of UC Irvine and Duke University. It received explicit approval by the UC Irvine Institutional Review Board. The cultures were plated on PEI (polyethyleneimine solution) substrate and maintained in Neurobasal media supplemented with B27 as described [2,5].

For RT-qPCR, the cell pellets were collected after treatments with vehicle (0.1% DMSO) or KP (50, 100, and 400nM) for 48h starting at day 6 in vitro.

For immunostaining, the cultures were treated for 48h with vehicle or 400 nM KP, fixed with 4% PFA at day 10, and processed for double-staining with anti-KCC2 and anti-synaptophysin or anti-NeuN (see antibody Supplementary Table 2). The 0.34μm confocal slices through entire cell layers at different optical fields (n = 17-28 for each group) of the fixated cultures were acquired using a Zeiss LSM700 confocal microscope.

Image Z-stacks were analyzed using Imaris 9.2.1 software. Optical density intensity sum values for each stack/channel were divided by corresponding data volumes and the resulting values were normalized to the number of specifically labeled cells within each stack (n=20-30). There were no significant differences in the number of cells between vehicle and KP-treated groups.

δ-catenin DNA constructs, transgenesis vectors

Plasmid containing human δ-catenin (CTNND2, NM_001288717) open reading frame was obtained from GeneCopeia (EX-A4285-M02) and cloned into pCMV-ENTER vector (Origene PS100001). Site-directed mutagenesis using Phusion DNA Polymerase enzyme (Thermofisher F549L) in conjunction with complementary primers bearing the specific mutation were used to generate the S276A δ-catenin mutation S276A. PCR was followed by Dpn1 enzyme digestion to remove parental plasmid DNA. All constructs were verified by sequencing. pCS-CMV-tdTomato plasmid was obtained from Addgene (cat. #30530).

Plasmid pAAV-hSyn-eNpHR 3.0-EYFP from Addgene (26972) was cut with Age I and Hind III enzymes to excise the eNpHR 3.0-EYFP open reading frames. The control tdTomato open reading frame as well as the

wild-type and mutant delta catenin open reading frames were generated with Age I and Hind III ends by PCR and subsequently inserted into the Age I / Hind III digested pAAV-hSyn plasmid. Orientation and sequence fidelity in the final constructs were verified by PCR and sequencing. AAV9 particles were packaged by the Duke University Viral Vector Core facility and were used at a titer of 10^{12} viral genome copies per mL.

Kcc2 promoter luciferase reporter assays

A fragment of the mouse *Kcc2* gene promoter (position −2052kbp to +476kbp) was amplified from genomic DNA prepared from cultured mouse primary glial cells. A 2.5kb PCR fragment was cloned into the pGL4.17-Basic Vector (Promega) to generate the *Kcc2* promoter reporter construct. TCF and Kaiso binding sites were identified in this fragment. Using wild-type construct pGL4.17-*Kcc2* as a template, site-directed mutagenesis using Phusion DNA Polymerase enzyme (Thermo Fisher F549L) in conjunction with complementary primers bearing the specific mutation were used to mutate the Kaiso and TCF DNA-binding sites. PCR was followed by Dpn1 enzyme digestion to remove parental plasmid DNA. All constructs were verified by sequencing.

N2a cells were grown to 90% confluency in 24-well dishes in 0.4 mL of medium (DMEM, 2% Fetal Bovine Serum, 2 mM glutamine, 1% Non-essential amino acids, and 1% Penicillin/Streptomycin). Cells were transiently transfected using TurboFect reagent (Thermo Fisher R0531), with 500 ng of the pGL4.17-constructs plus 20 ng of the control Renilla plasmid (Promega, E2231) to normalize for transfection efficiency. Twenty-four hours after transfection, luminescence was measured using the Dual-Luciferase® Reporter Assay System (Promega) in a microplate luminometer (Veritas, Turner Biosystems). Mutant promoters were compared to WT, and the response of the respective promoter to KP (400 nM) was measured. Three independent transfection experiments were carried out and LUC assays were done in triplicates for each transfection. RLU is expressed as firefly luciferase activity relative to Renilla LUC activity.

RT-qPCR

Total RNA was isolated from cultured cell samples using Directzol RNA miniprep kit (ZymoResearch). The protocol includes DNAse digestion to exclude genomic DNA from preparations. Total RNA (1µg)was reverse transcribed using oligo primers (dT) and SuperScriptIII first-strand synthesis kit (Invitrogen). Gene expression was assessed by quantitative real-time PCR using 2× SYBR Green Master Mix (Qiagen) and a three-step cycling protocol (anneal at 60°C /elongate at 72°C, denature at 95°C). Specificity of primers was verified by dissociation/melting curve for the amplicons when using SYBR Green as a detector. All reactions were performed in triplicates. The amount of target messenger RNA (mRNA) in the experimental group relative to that in the control was determined from the resulting fluorescence and threshold values (Ct) using the ΔΔCt method. β_{III}-tubulin was used as housekeeping gene. All PCR primers shown in Supplementary Table 1.

Behavioral assessments

For pain-related behavior, mechanical allodynia was assessed with von Frey filaments (Ugo Basile, Italy). Animals were habituated to the testing environment daily for at least 2 days before baseline testing. The room temperature and humidity remained stable for all experiments. The mice were placed on a 5×5-mm wire-mesh grid floor in individual compartments to avoid visual stimulation and allowed to adapt for 0.5 h prior to the von Frey test. The von Frey filament was then applied to the middle of the plantar surface of the hind paw, perpendicularly, with a series of von Frey hairs with logarithmically increasing stiffness (0.02–2.56g, Stoelting). The withdrawal responses following the hind paw stimulation were measured at least three times. We determined the 50% paw withdrawal threshold by up-down method [6].

For assessment in rotarod (RR), all animals received training prior to experiments; mice were placed on the RR apparatus set in an accelerating rotational speed mode (3–30 rpm, 300 s max) per trial. Following training, the average time to fall from the rotating cylinder over three trials was recorded as baseline latency (4-40rpm, 300s max/trial). Mice were injected daily with either vehicle or drug compounds before RR tests. Latency to fall was measured (4-40rpm, 300s max/trial (inter-trial interval is at least 15 min). The average latency to fall from the rod was recorded for each animal.

Conditioned place preference (CPP) was conducted using a CPP box, which consists of two conditioning chambers distinguished by visual and sensory cues, along with a small buffering chamber. All mice received a 3-day preconditioning habituation period with free access to both conditioning chambers and the time spent in each chamber was recorded for 15 min on day 3 after habituation. On conditioning days (day 4-10), mice first received the vehicle control (i.p. 5% DMSO, 5%Tween-80 in normal saline) paired with a randomly chosen chamber in the morning. After 4 hours, mice received KP (i.p. 30mg/kg) or vehicle, paired with the other chamber. During the conditioning, mice were allowed to stay only in the paired chamber for 15 min without access to other chambers. On test day (d11), mice were placed in the buffering chamber with free access to both conditioning chambers and choice behavior was recorded for 15 min. The CPP scores were calculated as post-conditioning time minus preconditioning time spent in the paired chamber.

Bone cancer pain model

Murine lung carcinoma cell line LLC1 (ATCC CRL-1642) was digested with 0.25% trypsin and suspended at 5×10^7/ml cells in PBS. Following previous protocol [7], mice were anesthetized with 3% isoflurane (oxygen flow: 1.0 L/min). The left leg was shaved, and the skin was disinfected with 10% povidone-iodine and 75% ethanol. A 0.5-1cm superficial incision was made near the knee joint to expose the patellar ligament. Then a 25-gauge needle was inserted at the site of the intercondylar notch of the left femur into the femoral cavity and the needle was then replaced with a 10 μL microinjection syringe containing 4 μL suspension of tumor cells (2×10^5) and 2 μL absorbable gelatin sponge solution for closure of the injection site. The contents of the syringe were slowly injected into the femoral cavity (2 min). To further prevent leakage of tumor cells from the bone's cavity, the injection site was sealed with silicone adhesive at the periost level. Animals with surgery related movement dysfunction or with mis-targeted tumor cell injection were excluded.

Radiography of bone imaging

Osteolytic bone destruction was assessed by Faxitron (Faxitron Bioptics, Tucson, Arizona). Radiographs of tumor-bearing femora were rated following a 0-5 score scale as previously described [7,8] by blinded readers: 0, normal bone without signs of destruction; 1, one to three radiolucent lesions indicative of bone destruction; 2, increased number of lesions (three to six lesions) and loss of medullary bone; 3, loss of medullary bone

and erosion of cortical bone; 4, full-thickness unicortical bone loss; 5, full-thickness bicortical bone loss and displaced skeletal fracture.

Chromatin immunoprecipitation

ChIP assay was carried out as described previously [1,2]. Primary cortical neurons (0.7 × 10^6) were used for each ChIP experiment. Cells were crosslinked with 1% formaldehyde for 30 min, washed twice with cold PBS, resuspended in lysis buffer [1%SDS, 10 mm EDTA, and 50 mm Tris-HCl, pH 8.0, with protease inhibitor cocktail (Roche)], and sonicated for 15 s pulses. The lysates were clarified by centrifugation at 10,000 rpm for 10 min at 4°C in a microcentrifuge. One-tenth of the total lysate was used as input control of genomic DNA. Supernatants were collected and diluted in buffer (1% Triton X-100, 2 mm EDTA, 150 mm NaCl, 20 mm Tris-HCl, pH 8.0, and protease inhibitor cocktail) followed by immunoclearing with 1 mg of salmon sperm DNA, 10 ml of rabbit IgG, and 20 ml of protein A/G-Sepharose (Santa Cruz Biotechnology) for 1h at 4°C. Immunoprecipitation was performed overnight at 4°C with 2 mg of each specific antibody. Precipitates were washed sequentially for 10 min each in TSE1 buffer (0.1% SDS, 1% Triton X-100, 2 mm EDTA, 150 mm NaCl, and 20 mm Tris-HCl, pH 8.0), TSE2 (TSE1 with 500 mm NaCl), and TSE3 (0.25 m LiCl, 1% NP-40, 1% deoxycholate, 1 mm EDTA, and 10 mm Tris-HCl, pH 8.0). Precipitates were then washed twice with 10 mm Tris/0.1 mm EDTA, pH 7.8 and extracted with 1% SDS containing 0.1 m $NaHCO_3$. Eluates were pooled and heated at 65°C for 4 h to reverse formaldehyde crosslinking. DNA fragments were purified with Qiagen Qiaquick kit. For ChIP PCR, 1 μl of a 25 μl DNA extraction was used.

Immunocytochemistry of cultured neurons

Immunocytochemistry labeling of cultured neuronal cells was carried out as previously described [1-3]. Primary antibodies are shown in Supplementary Table 2. Anti-KCC2 primary antibodies were validated as shown in Supplementary Fig. 2a. Secondary antibodies used were goat anti-mouse IgG Alexa Fluor 594 (Invitrogen A11032) and goat anti-rabbit IgG Alexa Fluor 594 (Invitrogen A11012). DAPI stain was obtained from Sigma Aldrich (D9542). Stained cells were observed using an inverted confocal microscope (Zeiss LSM780).

We obtained stacks of images recorded at 0.35 µm intervals through separate channels with a 63x oil-immersion lens (NA, 1.40, refraction index, 1.45). Zen software (Zeiss) was used to construct composite images from each optical series by combining the images recorded through the different channels, and the same software was used to obtain Z projection images (image resolution: 1024 × 1024 pixels; pixel size: 0.11 µm). ImageJ was used for morphometry.

Morphometry of δ-cat or ß-cat abundance was conducted on single neurons of primary cortical neurons from rat, vehicle- vs KP-treated. The micrograph was analyzed in ImageJ, determining average density of the catenin-labeled neuron in their respective nuclear vs cytoplasmic compartment. These densities were background corrected and a ratio was formed, nuclear abundance vs cytoplasmic, for each cell. See Supplementary Fig. 6b.

Spinal cord immunohistochemistry

All mice were deeply anaesthetized with isoflurane and then transcardially perfused with ice-cold 4% paraformaldehyde in 0.1 M phosphate buffer, pH 7.4 (4% PFA). Dissected spinal cord samples were then post-fixed overnight in 4% PFA at 4 °C, cryoprotected in a 20% sucrose solution in PBS at 4 °C, frozen in Tissue-Tek OCT (Sakura), and stored at −80 °C until sectioning. Samples were sectioned at 20 µm using a cryostat (Microm HM 505N). The sections were blocked with 2% bovine serum albumin (BSA) in PBS with 0.3% Triton X-100 (Blocking solution) at room temperature for 1h. The sections were treated with primary antibody in blocking solution at 4°C overnight. The sections were washed three times followed by secondary antibody treatment at 4°C for 2 hours. Anti-KCC2 antibody was validated as described above for immuno-cytochemistry. Nissl stain was used as counterstain not regulated by peripheral injury or treatment with KP (ThermoFisher/Invitrogen cat# N21480). The goat anti-rabbit IgG Alexa Fluor 488 was obtained from Invitrogen (A-11008). Morphometry was conducted using ImageJ with region-of-interest Rexed laminae I-II.

Cultured neuron KCC2 protein abundance assay - virtual Western blot

The method was conducted following [9,10], loading 500µg/mLprotein for KCC2-immunodetection, and 200µg/mL for $ß_{III}$-tubulin immunodetection.

Drug Affinity Responsive Target Stability (DARTS) assay

Cultured primary rat cortical neurons were treated with either vehicle DMSO (0.1%) or 20 µM KP for 30h. Cells were lysed in ice-cold lysis buffer (Tris.Cl pH8 50mM, NaCl 150mM, NP40 0.5%, N-dodecyl-b-D-maltoside 0.5%, Phosphatase Inhibitor (Pierce #88667) and Protease Inhibitor (Roche #11836153001)). Protein concentrations were determined by Bio-Rad DC Protein Assay kit using bovine albumin as standard. All steps were performed on ice. Samples were warmed to room temperature and digested with pronase (final concentration 1:500) for 30 min at 30 °C. Digestion was halted using 0.5M EDTA. Only proteins not bound to KP were digested. The protein mixture was dialyzed using dialysis cassettes (Thermo Fisher 66203, 2K MWCO) and analyzed by LC-MS/MS method to identify proteins that are bound to KP, the latter step carried out in the Duke Proteomics Core Laboratory.

Molecular Dynamics simulation

The structure of human GSK3β in complex with inhibitory pyridines, as determined by X-ray crystallography (PDB code 6V6L) was employed for molecular modeling [11]. Prior to docking, the KP and GSK3β structures were prepared using AutoDock Tools 4 software [12]. Initially, to evaluate the validity of the docking program, we first performed the redocking of pyridine compounds to 6V6L structure coordinates of GSK3β. All analysis and visualization of the structure files were done using Chimera [13].

Molecular dynamics simulations (MDS) were performed on the protein-ligand complex of the docking output. The all-atom solvent-explicit MDS was performed on the complex using DESMOND software (Desmond Molecular Dynamics System v2018, DE Shaw Research, New York NY). The force-field OPLS_2005 was used for model generation and energy minimization of the protein-ligand complex. The protein-ligand system was immersed in the orthorhombic predefined simple-point-charge water box model. The isothermic-isobaric NPT ensemble was used with 300 K temperature and 1.01325 bar pressure.

The MDS of the complex was carried out for 1000 nanosecond (ns) and 10000 trajectory structures were recorded. Clustering of 10000 trajectory structures into 100 clusters based on atomic root-mean-square deviation (RMSD) was performed using the DESMOND's clustering tool. The ensemble structure from the largest cluster was chosen for subsequent 100 ns simulations for the confirmation of stability.

Kinome analysis

Cultured rat primary cortical neurons were treated with either vehicle DMSO (0.1%) or 1 µM KP for 1h/24h. Cells were lysed in non-detergent-containing buffer (Tris.Cl pH8 50mM, NaCl 150mM, 0.5% Phosphatase Inhibitor (Pierce #88667) and Protease Inhibitor (Roche #11836153001)). 500 µL was removed and solid urea was added to a final concentration of 8M. Samples were sonicated for further solubilization. After clearing of insoluble material by centrifugation, protein concentration was measured by Bradford assay. 250 µg of total protein was removed from each sample and solubilization buffer was added to normalize all samples to 0.93 µg/µL protein. Samples were then spiked with bovine alpha-casein to 30 fmol/µg of total protein. Samples were reduced with 10 mM DTT at 32 °C for 45 min and then alkylated with 20 mM iodoacetamide at room temperature for 30 min. Samples were trypsin digested at 1:25 (enzyme-to-protein) overnight at 32 °C. Following acidification with TFA to pH 2.5, samples were subjected to a C18 solid-phase extraction cleanup. Eluted peptides were split 80% for phosphopeptide analysis and 20% reserved for unbiased differential expression. The phosphopeptide fraction (200 µg) was then frozen and lyophilized prior to phosphopeptide enrichment.

TiO2 Enrichment: Samples were resuspended in 65 µL of 1M glycolic acid in 80% MeCN/1% TFA and were enriched on TiO2 resin using a 10 µL GL Sciences microliter TiO2 spin tips following an established protocol (http://www.genome.duke.edu/cores/proteomics/samplepreparation/ documents/GL_SpinColumnProtocol_bmr_ejs_mt_061713.pdf). After elution and acidification, samples were lyophilized to dryness and resuspended in 100 µL of 0.15% TFA in water. After cleanup using a C18 STAGE tip, and resuspension in 2% acetonitrile, 0.1% TFA, 10 mM citric acid samples were quantified.

Quantitative analysis of Phosphopeptide Enriched Samples: Quantitative LC-MS/MS was performed in singlicate (4uL=33% of the total sample each injection) for phosphopeptide-enriched samples using a

nanoAcquity UPLC system (Waters Corp) coupled to a Thermo QExactive Plus high resolution accurate mass tandem mass spectrometer (Thermo) via a nanoelectrospray ionization source. Briefly, the sample was first trapped on a Symmetry C18 300 mm Å~ 180 mm trapping column for 6 min at 5l/min (99.9/0.1 v/v water/acetonitrile 0.1% formic acid), after which the analytical separation was performed on a 1.7 µm Acquity BEH130 C18 75 mm Å~250 mm column (Waters Corp). Peptides were held at 3% acetonitrile with 0.1% formic acid for 5 min and then subjected to a linear gradient from 3 to 30% acetonitrile with 0.1% formic acid over 90 min at a flow rate of 400 nL/min at 55˚C. Data collection on the QExactivePlus mass-spec was performed in a data-dependent acquisition (DDA) mode following protocol of the manufacturer.

Chloride imaging

We followed methodology described previously [1,3,14]. A Clomeleon expression plasmid was transfected into primary cortical neurons by electroporation (Amaxa Nucleofector Device). Transfected neurons were verified by yellow fluorescent protein (YFP) fluorescence, and ratiometric images (excitation at λ = 434 nm, dual emission at λ = 485 and 535 nm; for resting chloride, six stable frames at a rate 12 of per minute were captured, which were averaged) were acquired using RATIOTOOL. Calibration of Clomeleon signals (535 nm/485 nm emission ratio) was performed by using tributyltin-nigericin to establish a standard curve, which was then normalized for measured intraneuronal pH to take into account the pH sensitivity of Clomeleon.

Patch-clamp recordings in spinal cord slices

For spinal cord slice preparation, adult (5-7 weeks) male mice were anesthetized with urethane (1.5-2.0 g/kg, i.p.). The lumbosacral spinal cord was microsurgically removed and submerged into ice-cold dissection media which was saturated with 95% O_2 and 5% CO_2 at room temperature. After extraction and still under anesthesia, animals were euthanized. Transverse slices (300-400 µm) were cut using a vibrating microslicer (VT1200s Leica). The slices were incubated at 32°C for at least 30 min in regular artificial cerebrospinal fluid (aCSF), equilibrated with 95% O_2 and 5% CO_2.

The following solutions were used: Dissection solution: Sucrose 240 mM, $NaHCO_3$ 25 mM, KCl 2.5 mM, NaH_2PO_4 1.25 mM, $CaCl_2$ 0.5 mM, $MgCl_2$ 3.5 mM [15]. Regular artificial cerebrospinal fluid (ACSF): NaCl 117 mM, KCl 3.6 mM, $MgCl_2$ 1.2 mM, $CaCl_2$ 2.5 mM, $NaHCO_3$ 25 mM, NaH_2PO_4 1.2 mM, glucose 11 mM. The pH value of ACSF or dissection solution was adjusted to 7.4 when saturated with the gas. Normal intrapipette solution (pH 7.2 and 310 mOsm): K-methylsulfate 115 mM, KCl 25 mM, $MgCl_2$ 2 mM, HEPES 10 mM, GTP-Na 0.4 mM and Mg-ATP 5 mM.

Electrophysiological recordings were conducted as follows. A slice was placed in the recording chamber and completely submerged and superfused at a rate of 2-4 ml/min with aCSF saturated with 95% O_2 and 5% CO_2 at room temperature. Perforated patch-clamp was used to avoid alteration of the [Cl-]i. To measure the chloride equilibrium potential (E_{Cl}), gramicidin D (80 µg/mL with 0.8% DMSO final concentration, from an 8 mg/mL stock in DMSO) was added to the intrapipette solution, and 6-cyano-7-nitroquinoxaline-2,3-dione (CNQX, 10 µM), D_L-2-amino-5-phosphonovaleric acid (APV, 50 µM), and tetrodotoxin (TTX, 0.5 µM) were added to the aCSF solution. The tip of the patch pipette was filled with the normal intrapipette solution while the rest of the pipette contained the gramicidin-containing solution. After forming a seal on the membrane, we waited ~30 min for the gramicidin to induce sufficient cation-selective pores in the membrane and lowered the series resistance to below 100 MΩ. Membrane potential measurements were corrected for liquid junction potential, which was measured as in [16]. GABA (1 mM) was puffed locally and instantaneously, and the puff pipette was aimed toward the recording pipette. To determine the reversal potential of GABA-evoked currents, voltage ramps were applied from +8 to -92 mV over 200 ms at a holding potential of -42 mV. Since the voltage ramp might elicit a basal current, a control voltage ramp was applied, and 1 min later GABA was puffed followed by another voltage ramp [17]. The reversal potential was analyzed as in [17].

Signals were acquired using an Axopatch 700B amplifier and analyzed with pCLAMP 10.3 software. Only neurons with resting membrane potential < −50 mV and stable access resistance were included.

Supplementary Table 1: PCR Primers

Target	Forward primer	Reverse primer
**Site-directed mutagenesis**		
S276A	5'GCGCCCCAGGGCGGTGCACCCACCAAGCTGCAGCG3'	5'CGCTGCAGCTTGGTGGGTGCACCGCCCTGGGGCGC3'
Kaiso1	5GGGTTCTAGACTGAAACTAGTGACTCATTGGCTTTGTG'3'	5'CACAAAGCCAATGAGTCACTAGTTTCAGTCTAGAACCC3'
Kaiso2	5'GCTCAACAACCTGACGACTAGTGAGGACGGCGATGGGG3'	5'CCCCATCGCCGTCCTCACTAGTCGTCAGGTTGTTGAGC3'
TCF	5'CAAATCCCTTAGAAGCAACTAGTCGTCCATCGAAGAAGAC3'	5'GTCTTCTTCGATGGACGACTAGTTGCTTCTAAGGGATTTG3'
**RT-qPCR**		
Kcc2	5'CTGACGGACTGCGAGGACGG3'	5'GGCTGGTGTCCATCTCCTCCTCAA3'
tubulin	5'CCTGCCTTTTCGTCTCTAGCCGC3'	5GCTGATGACCTCCCAGAACTTGGC'3'
**ChIP Assay**		
Kaiso1	5'AGCTCATCCCATACTCAAACCCTG3'	5'GCATCTTGGAGATCTAAACTGCTAGC3'
Kaiso2	5'TGCATACGGGATGAGGTGAGCAGC3'	5'CAGAACCGTGGACAGCGCCTAGCG 3'
TCF	5'CTGAGCTGTATATCACACGGTCTGC3'	5'TACGCTACCCAGCTGTCTCTGATTC3'

Supplementary Table 2: Primary antibodies

Antibody	Source	purpose	dilution
anti-KCC2	Millipore (07-432), C-terminal immunogen	ICC, IHC	1:400 (ICC), 1:2000 (IHC)
anti-KCC2	Novus NBP1-74063, N-terminal immunogen (identical to LS Bio LS-C135150)	micro-elpho	1:1000
anti-$ß_{III}$-tubulin	Abcam (mouse, ab78078; rabbit, ab229590)	ICC	1:800
anti-$ß_{III}$-tubulin	Cell Science (rabbit mAb; 2128S)	micro-elpho	1:2000
anti-ß-catenin	SigmaAldrich (PLA0230)	ICC	1:400
anti-δ-catenin	SigmaAldrich (MABN2254)	ICC	1:400
anti-Synaptophysin	ThermoFisher (MA1-213)	ICC	1:200
anti-FLAG	SigmaAldrich (F3165)	ICC	1:1000
anti-NeuN	BioLegend (834501)	ICC	1:500

Supplementary Figures

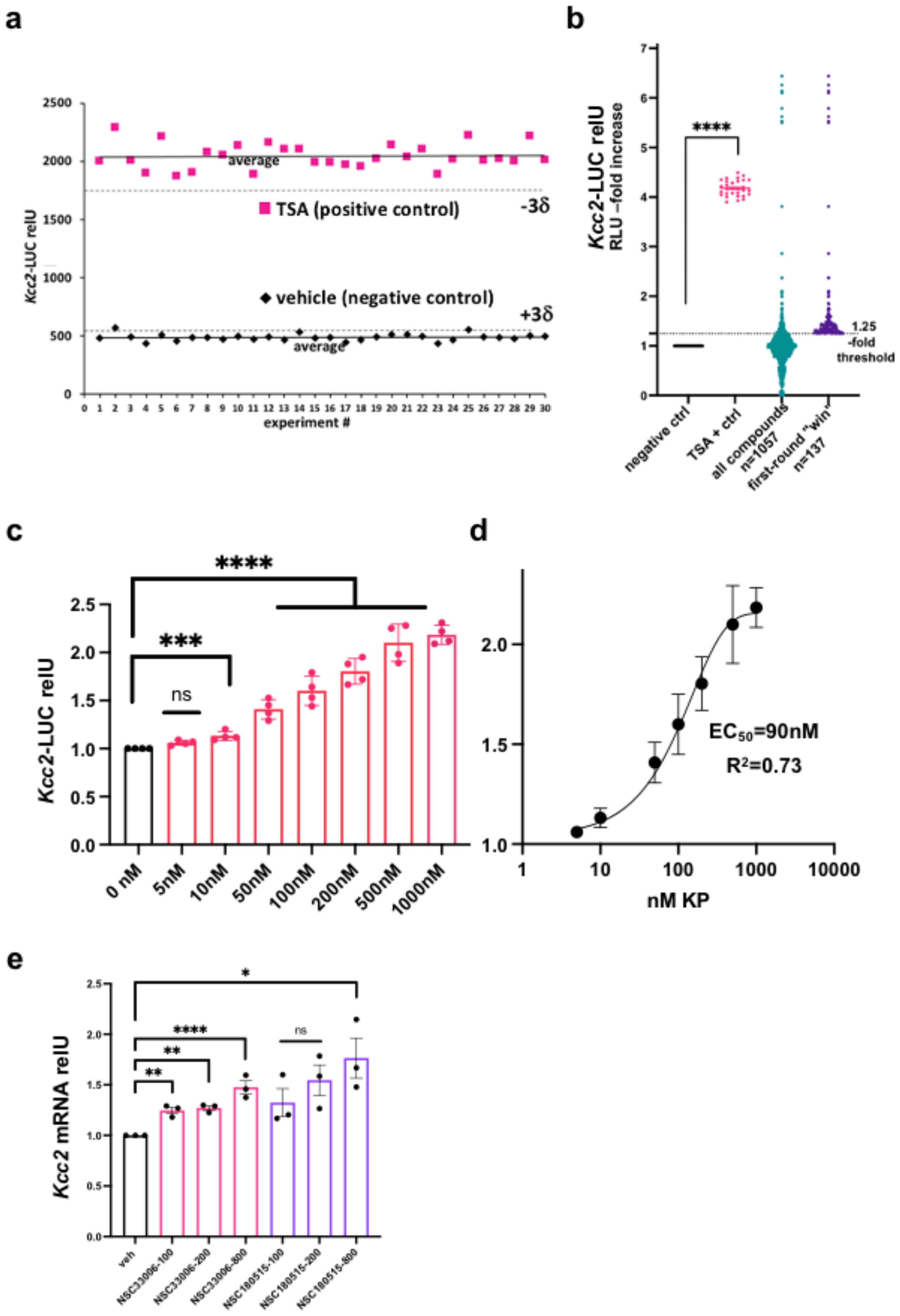

Supplementary Fig. 1. Screen-related Supplementary Findings

a) Average positive and negative control *Kcc2*-LUC measurements obtained for the 30 screening assays that were conducted to assess the 1057 compounds. Note positive controls (pink) consistently yielding approximately 2000 RLU, negative control (black) 500 RLU, 3δ lines dotted below and above the respective average (full line).

b) Dot-plot of the data from a), with negative control pegged to "1" (black), resulting in a -fold increase of the positive control of 4.1-fold (pink), highly significantly increased, ****$p<0.0001$, two-sided t-test. Next, green and purple dot-clouds indicate metrics of all 1057 compounds (green), and next to it the 137 first-round "winner" compounds (purple) with a -fold increase ≥1.25-fold.

c) *Kcc2*-RLU readout as a function of increasing doses of KP, applied to mouse primary cortical neurons, as for Fig. 1, KP treatment on DIV3, readout on DIV5, note significantly increased acitivity of the *Kcc2* promoter starting at 10nM concentration KP; concentrations beyond 1µM did not further enhance activity. Data are represented as mean values +/-SEM. n=4 independent neuronal cultures per concentration. ***$p=0.0002$; ****$p<0.0001$, one way ANOVA.

d) Dose-response curve, from c), indicating an EC50 of 90nM. Data are represented as mean values +/-SEM. Pearson correlation (two-sided analysis) was significant $p=0.0069$, $R^2=0.73$.

e) Significant increase in *Kcc2* mRNA abundance in rat primary cortical neurons in response to treatment with primary screen "hit" compounds, NSC33006 (for all three concentrations tested, 100, 200 and 400nM), and NSC180515 (only significant increase at 400nM). Data are represented as mean values +/-SEM of *Kcc2* mRNA expression after compound treatment, which was started at DIV5, cells harvested on DIV8. n=3 independent neuronal cultures, *$p=0.0134$, **$p=0.0045$, $p=0.0025$, ****$p<0.0001$, one way ANOVA

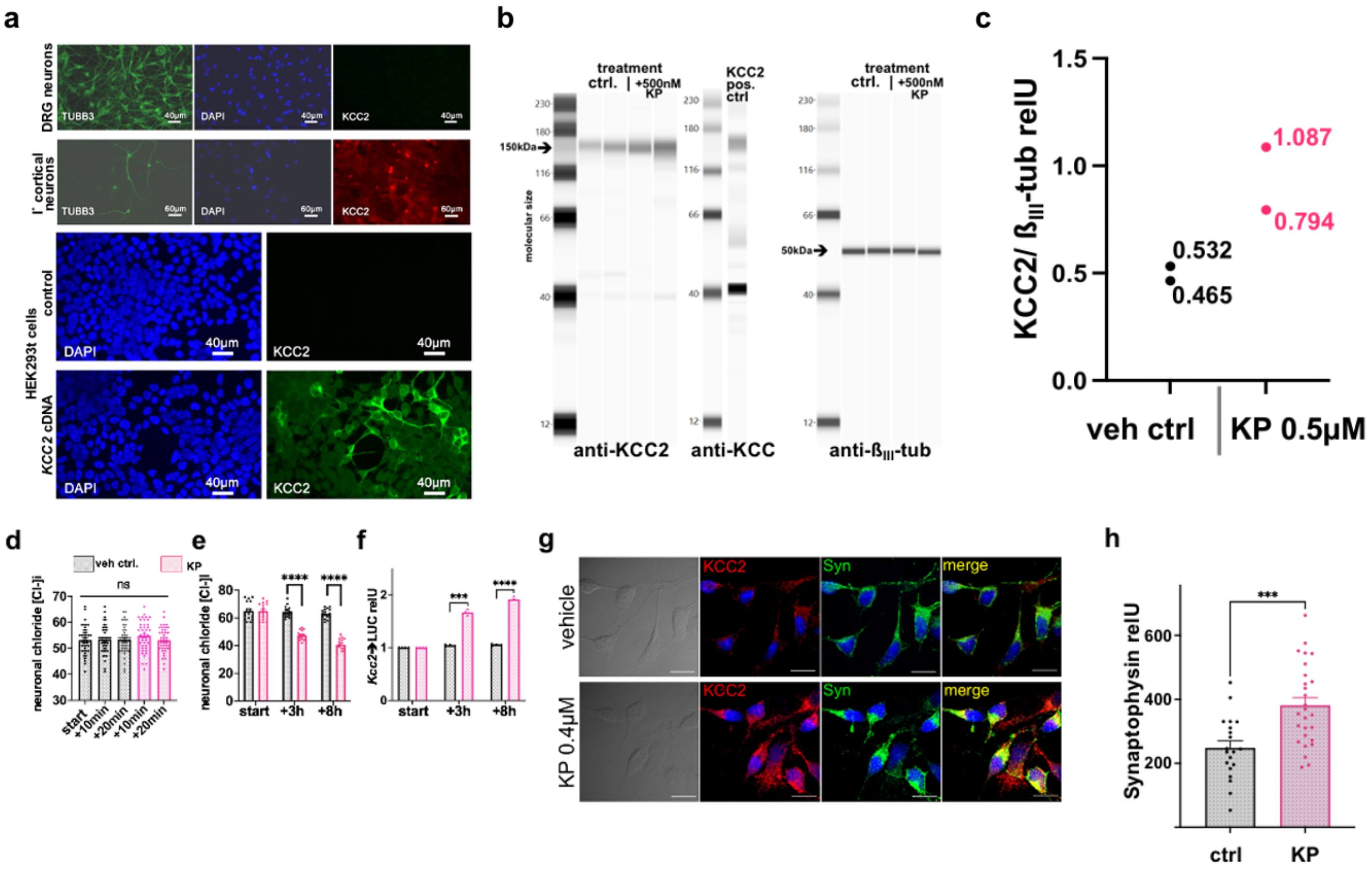

Supplementary Fig.2. Kenpaullone evokes increased KCC2 protein expression; Kenpaullone does not enhance KCC2 chloride transporter-mediated chloride efflux

a) Validation of anti-KCC2 antibody (MIllipore 07-432, raised against C-terminus, reported in [18,19]) used in immunocytochemistry and immunohistochemistry. Upper panels: note positive signal (red) in cortical neurons at DIV8, complete absence thereof in cultured DRG neurons DIV5; green channel: ICC ß$_{III}$-tubulin, blue channel: nuclear stain with DAPI Lower panels: HEK293t cells, transfected with human *KCC2* cDNA or control transfection (empty vector). Secondary antibody with green fluorescence, clear +/- pattern when comparing presence of KCC2 labeling in KCC2 transfected cells (lower panel) vs absence thereof in control-transfected cells (micrograph above). Cells fixated 48h after transfection, then 2-step immunodetection. Representative micrographs derived from 4 independent neuronal cultures for primary neurons, 4 independent transfections of HEK293t cells.

b) Increased protein abundance of KCC2 in primary neurons treated with KP. Electrophoretic size separation of protein extract from rat primary cortical neuronal cultures, treated with 0.5µM KP or vehicle control from DIV5-DIV8 (2 large independent primary neuronal cultures, split evenly, then treated with KP or vehicle

control), in microcapillaries, subsequent immunodetection (using previously validated N-terminal KCC2 antibody, see Methods and Supplementary Table 2) using chemoluminescence. Left-hand side pherogram-virtual blot showing KCC2 migrating at 150kDa, also present in positive control from mouse brain. Note increased signal in two right-hand side lanes of KP-treated neuronal cultures. Right-hand side pherogram shows house-keeping protein, neuronal $ß_{III}$ tubulin (50kDa) with equal abundance in all 4 cultures, which was used for normalization of the KCC2 densitometric measure.

c) Quantification of the KCC2 signal, normalized for $ß_{III}$-tubulin signal, lanes from panel b) showing increased abundance in KP-treated cultures.

d) Rat primary cortical neurons, n=42 neurons/group. Data are represented as mean values +/-SEM. No difference was observed in neuronal chloride levels, measured with clomeleon fluorescent indicator (main ms., Fig. 2d-e) after KP treatment (at DIV5, 0.5μM) vs vehicle at the 10 min and 20 min time-points.

e) Rat primary cortical neurons, clomeleon-based measurement of [Cl-]i, n=16 neurons/group. Data are represented as mean values +/-SEM. Note significant decrease of [Cl-]i at the 3h time-point after treatment with KP (at DIV5, 0.5μM), more pronounced at 8h. ****p<0.001, 2-way ANOVA.

f) Rat primary cortical neurons, transfected with red-shifted luciferase, driven by *Kcc2* promoter, as in [1]. n=4 independent cultures. Data are represented as mean values +/-SEM. When treating with KP (0.5μM at DIV5), note significantly increased activation of the *Kcc2* promoter 3h after start of treatment, more pronounced at 8h. ***p=0.0001, ****p<0.0001, 2-way ANOVA.

g) Primary human fetal cortical neurons; treatment with 0.4μM KP, as in main manuscript Figure 2f. Representative confocal images at DIV10 co-immuno-labelled for KCC2 (red) and synaptophysin (green), based on 3 independent neuronal cultures resulting in a total of 20 confocal slices for vehicle control and 27 confocal slices for KP-treated, also note the bright-field image (left-hand) and nuclear stain (DAPI - blue fluorescence), right-hand side showing merged fluorescent micrographs. Note enhanced expression of synaptophysin and of KCC2 in response to KP, also co-localization of KCC2 and synaptophysin. Scalebar=10μm.

h) Primary human fetal cortical neurons, morphometry of synaptophysin ICC shows significantly increased expression (53%) vs vehicle after KP treatment (0.4μM, as in g)). n=20 confocal slices harboring 20-30 neurons per slice for control, n=27 confocal slices for KP treated cultures; ****p*=0.0003, two-sided t-test.

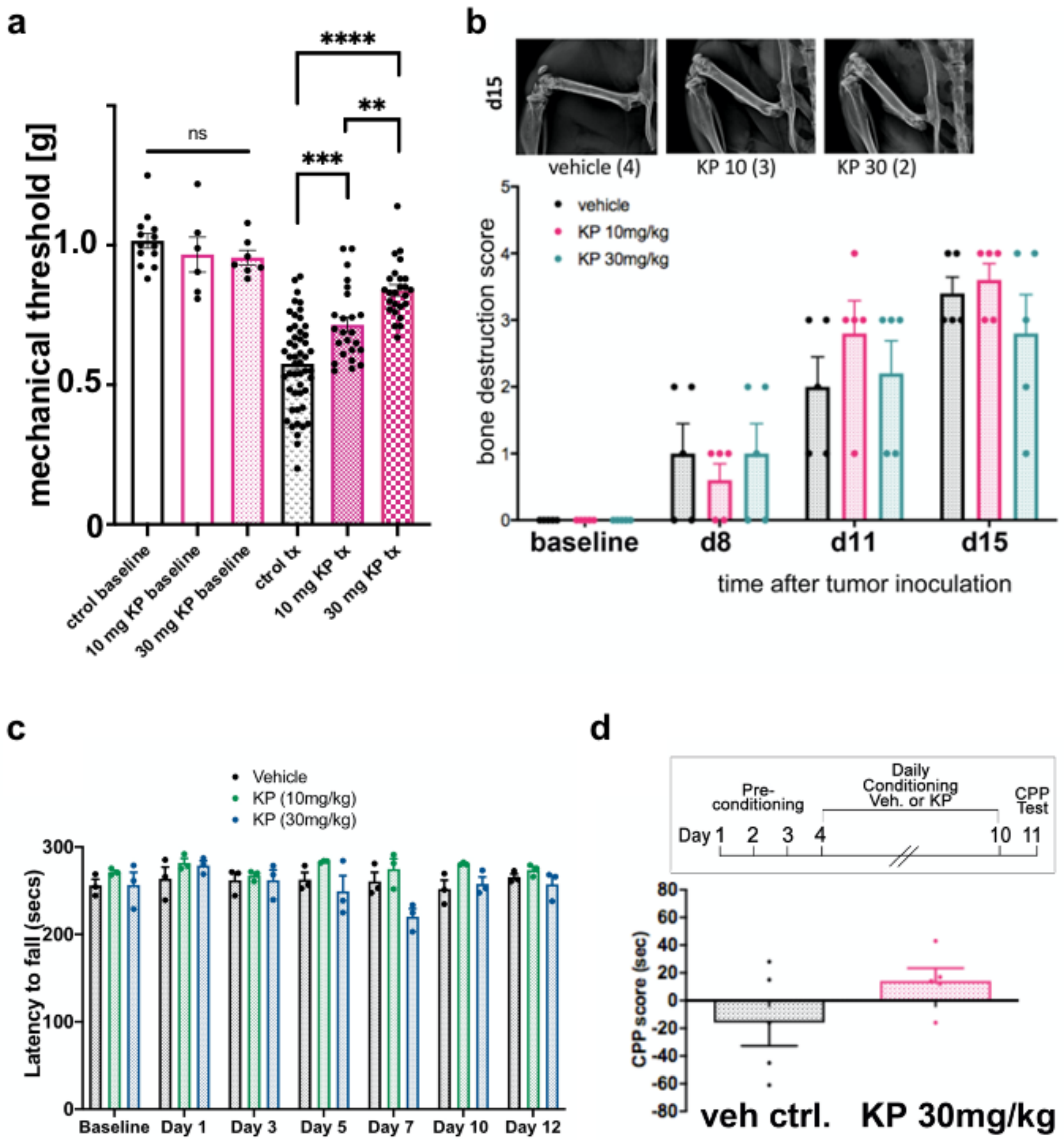

Supplementary Fig. 3. Kenpaullone - dose-dependent analgesia in nerve constriction neuropathic pain, lack of effect on osteolytic bone lesions, rotarod performance and conditioned place preference

a) Panel illustrating Fig. 3a of the main ms, showing statistically significant differences between control, 10 mg and 30 mg KP doses after all measurements from d4-14 were aggregated; statistic assessment was conducted using a repeated measures mixed effects model. Data are represented as mean values +/-SEM.

n per group were: base line - one data point per animal, n=13 (control), n=6 (KP 10 mg/kg), n=7 (KP 30 mg/kg); combined treatment - four data points per animal, n=50 (control), n=24 (KP 10 mg/kg), n=25 (KP 30 mg/kg). **p=0.0081, ***p=0.004 , ****p<0.0001, mixed model statistics.

b) KP did not contain growth of mouse LLC lung tumor cells infused into the femur

Osteolytic damage to femur (exemplars upper panel, at d15, for each group, with assessed severity) was not contained at any time point, not at 10 nor at 30 mg/kg bw KP. Data are represented as mean values +/- SEM.n=5 mice per group.

c) Effect of KP on mice' motor function and coordination – rotarod assay. Bar diagram showing the mean of elapsed time on the rotarod. KP did not affect motor stamina and coordination in mice, with the one-time exception of high-dose KP on day 7. Data are represented as mean values +/-SEM. n=3 mice per group.

d) KP does not evoke conditioned place preference (CPP). Timeline for CPP assay shown on top. Bottom: No significant difference in CPP scores between KP and vehicle. Data are represented as mean values +/- SEM. n=5 mice/group.

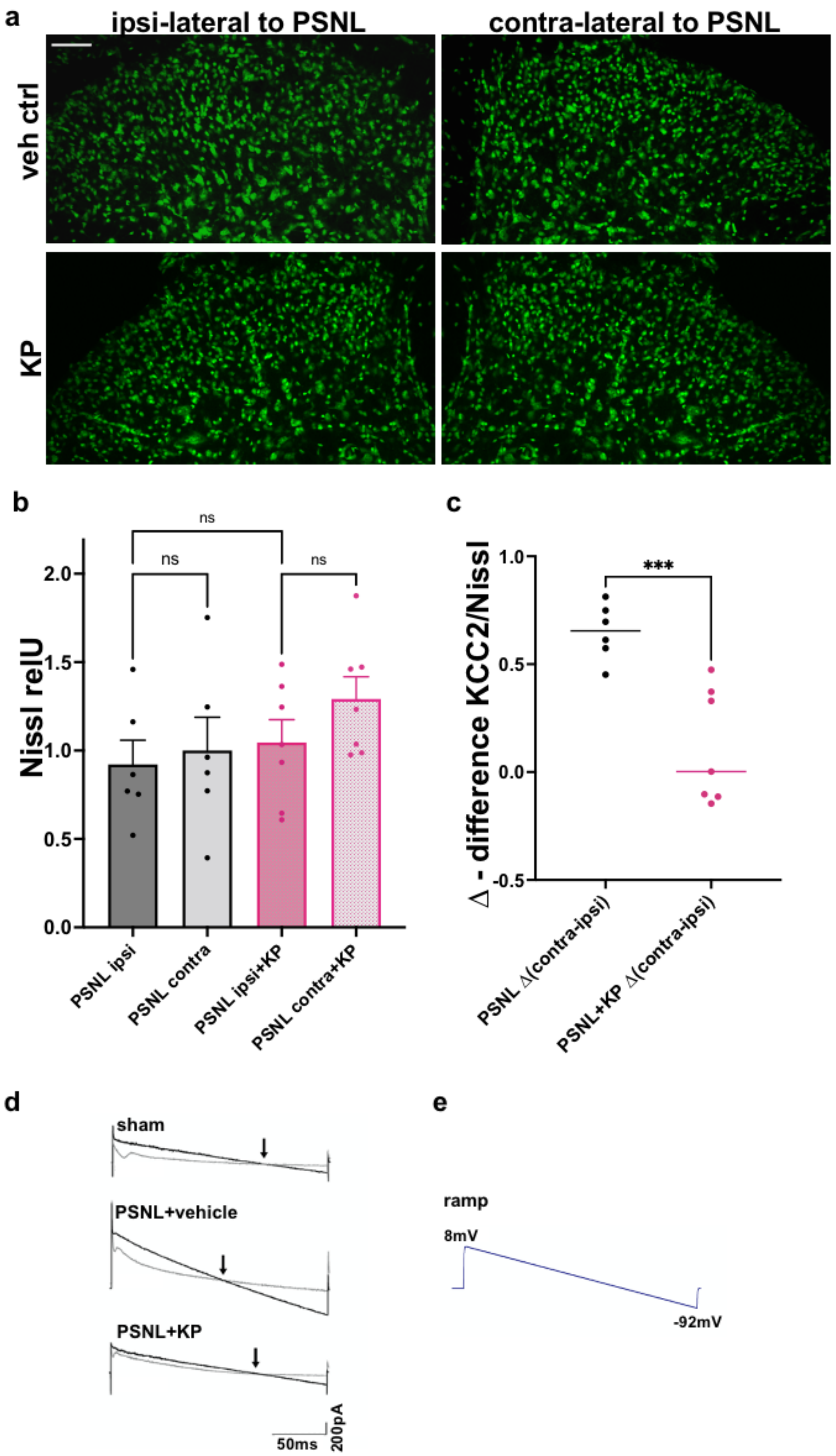

Supplementary Fig. 4. Electrophysiological recordings from spinal cord dorsal horn.

a) Mouse lumbar spinal cords, dissected at d7 after PSNL, treatment with 10 mg//kg KP daily after injury. Labeling with green fluorescent Nissl stain, see "Detailed Methods". Representative micrographs, 4

micrographs at spinal level L5 derived from each animal (n=6 vehicle control, n=7 KP-treated). Scale bar = 100 μm.

b) As in a), morphometric results of densitometry of the Nissl stain in laminae I-II ipsi- and contralateral to PSNL, with vehicle and KP treatment. Note absence of any statistically-significant difference between groups. Data are represented as mean values +/-SEM. n=6 (vehicle control), n=7 (KP-treated), one-way ANOVA.

c) Supplementary data to Fig. 4b (main manuscript). For each animal (n=6 vehicle control, n=7 KP-treated), the difference in expression level of KCC2 in layers I-II, normalized for Nissl stain, was calculated for contra-lateral side minus ipsi-lateral (to PSNL), and this difference was normalized to KCC2 expression measurement contralateral. This ratio goes up with high expression level of KCC2 contralateral and low expression level KCC2 ipsilateral; the ratio diminishes for high expression level KCC2 ipsilateral (taking into account similar levels of appreciable expression of KCC2 on the contralateral side, Fig. 4b). Each dot represents the value from one mouse, black - vehicle control group, purple - KP-treated group; solid line indicates the statistical mean for the respective group. Note the highly significant difference (***p=0.001) between vehicle- and KP-treated animals. n=6 (vehicle control), n=7 (KP-treated), two-sided t-test.

d) Current responses of lamina-II neurons to a voltage ramp from +8 to -92 mV (shown in panel **e**) in control (grey traces, obtained before GABA puff (1mM)), or at the end of a puff of GABA (black trace) in sham, PSNL+vehicle and PSNL+KP groups. Reversal potential of the GABA-evoked current is at the voltage where the grey and black traces intersect (arrow).

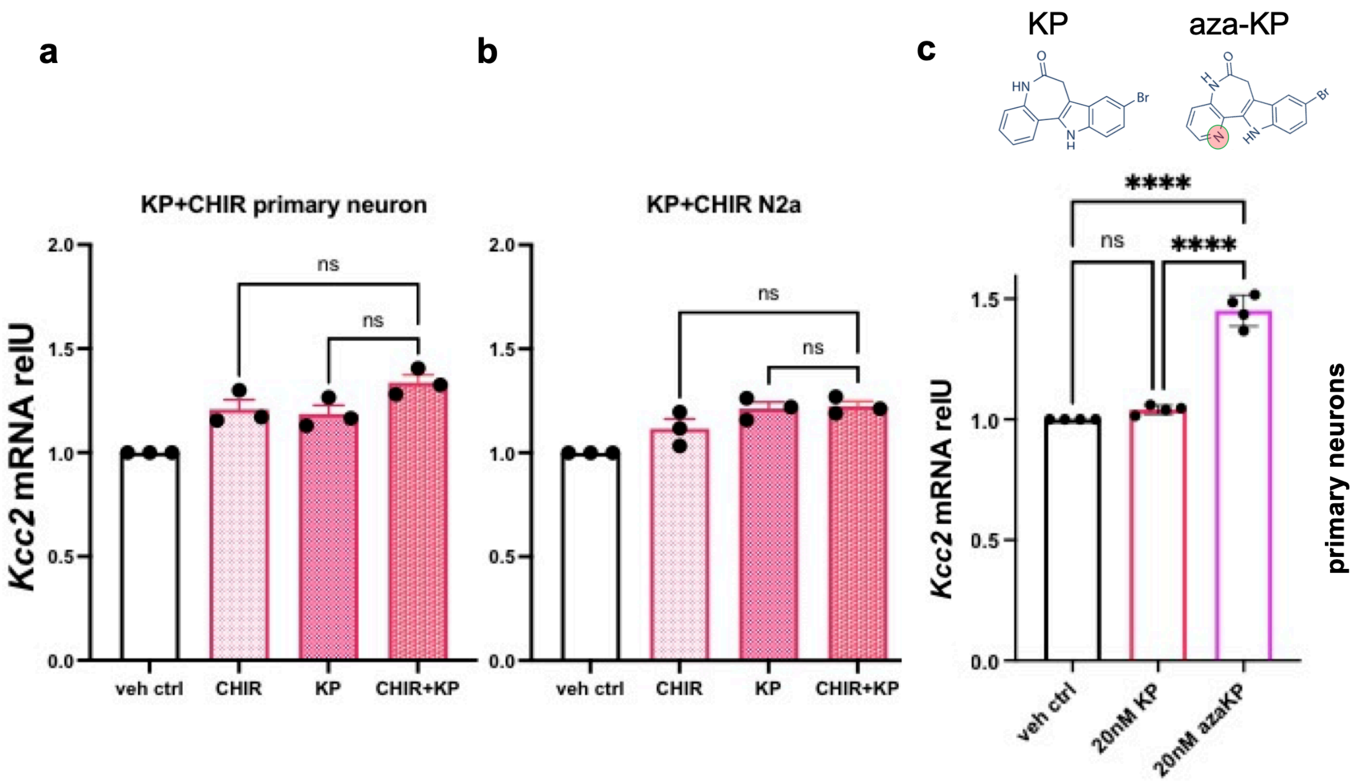

Supplementary Fig. 5: Ancillary GSK3-inhibitory compound findings

a) No significant increase in *Kcc2* mRNA abundance when co-treating primary cortical neuronal cultures with GSK3-inhibitory CHIR99201 and KP. Compound treatment DIV5-8, CHIR99201 at 10nM, KP at 0.5μM. Significant increase vs vehicle control for all three treatments (not indicated significance levels above bars), but of note there were no significant differences between co-treatment and individual treatments. Data are represented as mean values +/-SEM. n=3 individual rat cortical primary neuronal cultures per group; one-way ANOVA.

b) No significant increase in *Kcc2* mRNA abundance when co-treating differentiated N2a neural cells with GSK3-inhibitory CHIR99201 and KP. Data are represented as mean values +/-SEM. n=3 individual differentiated N2a neural cell cultures per group; one-way ANOVA. Compound treatment for 72h.

c) GSK3ß-inhibitory aza-KP increases *Kcc2* mRNA expression. Treatment with GSK3ß-inhibitory aza-KP at 20nM, which is 100-fold less potent to inhibit CDK than KP, evoked significant increase of *Kcc2* mRNA abundance, whereas 20nM KP did not significantly increase *Kcc2* mRNA in the same experiment, using rat primary cortical neurons, treated with compound DIV5-8. Data are represented as mean values +/-SEM. n=4 independent neuronal cultures; ****p<0.0001 one-way ANOVA.

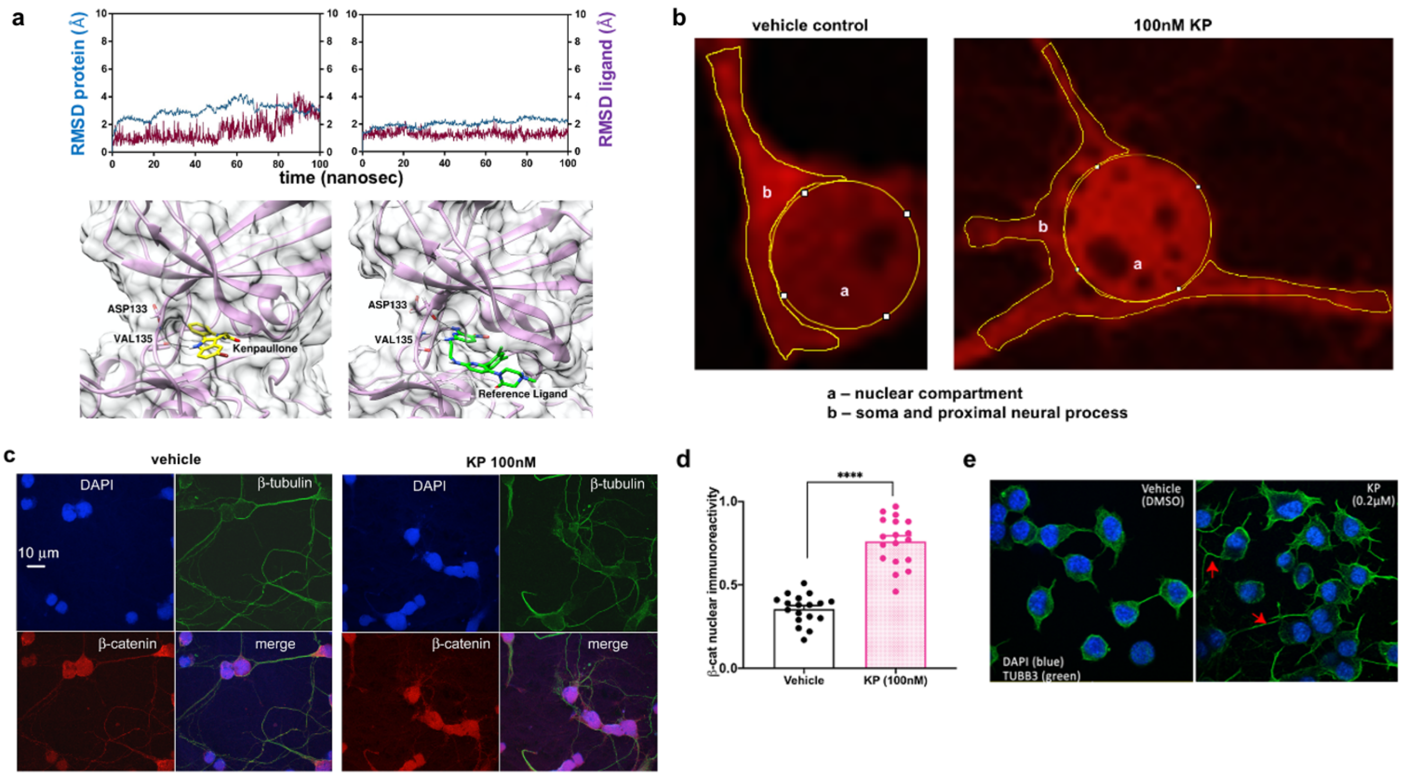

Supplementary Fig. 6. Kenpaullone binds GSK3ß, facilitates increased abundance of β-catenin in the neuronal nucleus, and enhances neuronal differentiation.

a) Upper panel, left hand: molecular dynamics simulation generated a candidate binding site of KP in the ATP binding pocket of human GSK3ß (pdb 6V6L), which again was simulated for 100 nanoseconds; y-axis shows root mean square distance (RMSD) in Angstrom (Å), left y-axis for GSK3ß (blue trace), and right y-axis for KP (magenta trace); note both remaining below 4Å.

Upper panel, right hand: comparable 100ns molecular dynamics simulation of a known GSK3ß inhibitor, "compound 2", which has previously been visualized by X-ray crystallography complexed with GSK3ß [11].

Lower panels: ATP binding domain of GSK3ß (see main Fig. 6a), with simulated KP binding and structurally-verified compound 2 binding, note Val135 as relevant GSK3ß residue to form hydrogen bonds with each compound that stabilize binding.

b) Schematic illustration of morphometry of δ-cat abundance of a single neuron (primary cortical neuron, rat), vehicle- vs KP-treated, treatment with 100nM KP from DIV5-8. Opening the micrograph of the immunolabeled cell in ImageJ, the nuclear compartment (defined by DAPI fluorescence in the blue channel) was demarkated using the ImageJ region tool, round or eliptical pattern (yellow outline). Then, using the freehand selection

mode, the soma-cytoplasmic compartment was indicated as well, guided by δ-cat fluorescence, and including ≤10μm neural process in case there were δ-cat fluorescent processes. Average density was determined, using ImageJ, in each compartment (a - nuclear, b - soma and proximal neural process), background-subtracted, and included into the ratio a/b ("nuclear density/density of soma and proximal neural process"), which was determined for each cell.

c) Micrographs: Representative immuno-labeling of neuronal β_{III}–tubulin (green) and β-catenin (red) before (left-hand panels) and after KP treatment (right-hand panels) in primary cortical neurons from rat.

DAPI stain is used to define nuclear compartment for morphometric assessment. Representative micrographs for n=18 neurons assessed per treatment arm, 4 independent primary neuronal cultures.

d) KP treatment (100nM DIV5-8) significantly increases β-catenin abundance into the nucleus (relative abundance, normalized for cytoplasmic abundance, as in b), see also Supplementary Methods). Data are represented as mean values +/-SEM. n=18 neurons/group. ****p<0.0001 two-sided t-test

e) Neuronalization of N2a cultured cells in response to KP (0.2μM for 3 days), note increased process formation and expression of neuronal β_{III}–tubulin (green; red arrows). Representative micrographs based on 4 independent cultures of neuronalized N2a cultures.

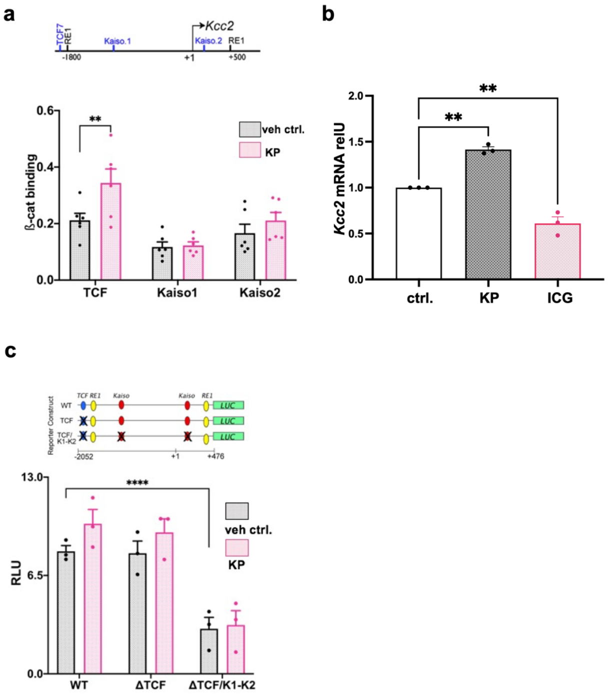

Supplementary Fig. 7. Kenpaullone increases β-catenin binding to *Kcc2* promoter

a) KP increases ß-cat binding to the TCF site in the *Kcc2* promoter.

Upper panel: Structure of mouse-*Kcc2* gene encompassing 2.5kb surrounding the TSS (+1), as in Fig. 7a. Bottom, bar diagram: Chromatin immuno-precipitation using anti-ß-cat antibody in primary rat cortical neurons (KP treatment at 100nM DIV5-8) reveals binding of ß-cat to TCF, to minor degree to Kaiso1, 2. KP significantly increases binding of β-cat to the *Kcc2* promoter on the TCF binding site. Data are represented as mean values +/-SEM. n=6 independent rat primary neuron cultures were ChIP-ed, **p=0.0041 two-sided t-test.

b) Rat primary cortical neurons, treated with KP (0.5μM) and catenin inhibitor ICG (0.5μM) DIV5-8. Note significant increase of *Kcc2* mRNA expression when treating with KP, significant reduction with ICG. Data are represented as mean values +/-SEM. n=3 independent neuronal cultures; **p=0.0011, p=0.0016 one-way ANOVA.

c) Relevance of TCF DNA binding site on regulation of *Kcc2*➔LUC

N2a differentiated cells were used as shown in Supplementary Fig. 6a (KP treatment at 100nM for 3 days). Top panel: mouse *Kcc2* promoter constructs, as in Fig. 7b. Bottom, bar diagrams: KP did not increase promoter activity for any of the constructs. Triple-deletion of TCF and both Kaiso sites rendered the *Kcc2* promoter very low in activity, vs WT (without KP), at <1/3 of its activity. Data are represented as mean values +/-SEM. n=3 independent cultures, **** p<0.0001 WT vs triple-deletion construct, 1-way ANOVA.

Supplementary References

1 Yeo, M., Berglund, K., Augustine, G. & Liedtke, W. Novel repression of Kcc2 transcription by REST-RE-1 controls developmental switch in neuronal chloride. *J Neurosci* **29**, 14652-14662 (2009).

2 Yeo, M. *et al.* Bisphenol A delays the perinatal chloride shift in cortical neurons by epigenetic effects on the Kcc2 promoter. *Proc Natl Acad Sci U S A* **110**, 4315-4320, doi:10.1073/pnas.1300959110 (2013).

3 Liedtke, W. *et al.* Highly conductive carbon nanotube matrix accelerates developmental chloride extrusion in central nervous system neurons by increased expression of chloride transporter KCC2. *Small (Weinheim an der Bergstrasse, Germany)* **9**, 1066-1075, doi:10.1002/smll.201201994 (2013).

4 Zhang, J. H., Chung, T. D. & Oldenburg, K. R. A Simple Statistical Parameter for Use in Evaluation and Validation of High Throughput Screening Assays. *Journal of biomolecular screening* **4**, 67-73, doi:10.1177/108705719900400206 (1999).

5 Pelsman, A. *et al.* GVS-111 prevents oxidative damage and apoptosis in normal and Down's syndrome human cortical neurons. *International journal of developmental neuroscience : the official journal of the International Society for Developmental Neuroscience* **21**, 117-124 (2003).

6 Chen, G., Park, C. K., Xie, R. G. & Ji, R. R. Intrathecal bone marrow stromal cells inhibit neuropathic pain via TGF-beta secretion. *J Clin Invest* **125**, 3226-3240, doi:10.1172/JCI80883 (2015).

7 Honore, P. *et al.* Osteoprotegerin blocks bone cancer-induced skeletal destruction, skeletal pain and pain-related neurochemical reorganization of the spinal cord. *Nat Med* **6**, 521-528, doi:10.1038/74999 (2000).

8 Yang, Y. *et al.* Delayed activation of spinal microglia contributes to the maintenance of bone cancer pain in female Wistar rats via P2X7 receptor and IL-18. *J Neurosci* **35**, 7950-7963, doi:10.1523/JNEUROSCI.5250-14.2015 (2015).

9 Onder, S. *et al.* Mass Spectral Detection of Diethoxyphospho-Tyrosine Adducts on Proteins from HEK293 Cells Using Monoclonal Antibody depY for Enrichment. *Chemical research in toxicology* **31**, 520-530, doi:10.1021/acs.chemrestox.8b00083 (2018).

10 Schopfer, L. M. *et al.* The C5 Variant of the Butyrylcholinesterase Tetramer Includes a Noncovalently Bound 60 kDa Lamellipodin Fragment. *Molecules (Basel, Switzerland)* **22**, doi:10.3390/molecules22071083 (2017).

11 Ramurthy, S. *et al.* Discovery and optimization of novel pyridines as highly potent and selective glycogen synthase kinase 3 inhibitors. *Bioorg Med Chem Lett* **30**, 126930, doi:10.1016/j.bmcl.2019.126930 (2020).

12 Morris, G. M. *et al.* AutoDock4 and AutoDockTools4: Automated docking with selective receptor flexibility. *Journal of computational chemistry* **30**, 2785-2791, doi:10.1002/jcc.21256 (2009).

13 Pettersen, E. F. *et al.* UCSF Chimera--a visualization system for exploratory research and analysis. *Journal of computational chemistry* **25**, 1605-1612, doi:10.1002/jcc.20084 (2004).

14 Kuner, T. & Augustine, G. J. A genetically encoded ratiometric indicator for chloride: capturing chloride transients in cultured hippocampal neurons. *Neuron* **27**, 447-459 (2000).

15 Cheng, L. *et al.* Identification of spinal circuits involved in touch-evoked dynamic mechanical pain. *Nature neuroscience* **20**, 804-814, doi:10.1038/nn.4549 (2017).

16 Jiang, C. Y., Fujita, T. & Kumamoto, E. Synaptic modulation and inward current produced by oxytocin in substantia gelatinosa neurons of adult rat spinal cord slices. *Journal of neurophysiology* **111**, 991-1007, doi:10.1152/jn.00609.2013 (2014).

17 Billups, D. & Attwell, D. Control of intracellular chloride concentration and GABA response polarity in rat retinal ON bipolar cells. *The Journal of physiology* **545**, 183-198 (2002).

18 Kontou, G. *et al.* KCC2 is required for the survival of mature neurons but not for their development. *The Journal of biological chemistry*, 100364, doi:10.1016/j.jbc.2021.100364 (2021).

19 Kelley, M. R. *et al.* Locally Reducing KCC2 Activity in the Hippocampus is Sufficient to Induce Temporal Lobe Epilepsy. *EBioMedicine* **32**, 62-71, doi:10.1016/j.ebiom.2018.05.029 (2018).
