# Supplementary material for: Repurposing cancer drugs identifies kenpaullone which ameliorates pathologic pain in preclinical models via normalization of inhibitory neurotransmission
Source: Nat Commun. 2021 Oct 27;12:6208. doi: 10.1038/s41467-021-26270-3 (PMC8551327; doi:10.1038/s41467-021-26270-3)
Supplement: Supplementary file 3 — Description of Additional Supplementary Files [file 41467_2021_26270_MOESM3_ESM.pdf]

Description of Additional Supplementary Files

Supplementary Data 1: Screening results of 1057 cell growth-regulatory compounds from 2 NCI compound libraries on metrics of Kcc2 gene expression

Supplementary Data 2: DARTS assay to discover binding of neuronal proteins to GSK3-beta

Supplementary Data 3: Kinome data of primary cortical neurons treated with Kenpaullone
