# Supplementary material for: Repurposing cancer drugs identifies kenpaullone which ameliorates pathologic pain in preclinical models via normalization of inhibitory neurotransmission
Source: Nat Commun. 2021 Oct 27;12:6208. doi: 10.1038/s41467-021-26270-3 (PMC8551327; doi:10.1038/s41467-021-26270-3)
Supplement: Supplementary file 7 — Reporting Summary [file 41467_2021_26270_MOESM7_ESM.pdf]

nature research

Corresponding author(s): Wolfgang Liedtke
Last updated by author(s): Sep 12, 2021

Reporting Summary

Nature Research wishes to improve the reproducibility of the work that we publish. This form provides structure for consistency and transparency in reporting. For further information on Nature Research policies, see Authors & Referees and the Editorial Policy Checklist.

Please do not complete any field with "not applicable" or n/a. Refer to the help text for what text to use if an item is not relevant to your study.
For final submission: please carefully check your responses for accuracy; you will not be able to make changes later.

Statistics

For all statistical analyses, confirm that the following items are present in the figure legend, table legend, main text, or Methods section.

n/a	Confirmed	
☐	☒	The exact sample size (*n*) for each experimental group/condition, given as a discrete number and unit of measurement
☐	☒	A statement on whether measurements were taken from distinct samples or whether the same sample was measured repeatedly
☐	☒	The statistical test(s) used AND whether they are one- or two-sided<br>*Only common tests should be described solely by name; describe more complex techniques in the Methods section.*
☒	☐	A description of all covariates tested
☐	☒	A description of any assumptions or corrections, such as tests of normality and adjustment for multiple comparisons
☐	☒	A full description of the statistical parameters including central tendency (e.g. means) or other basic estimates (e.g. regression coefficient) AND variation (e.g. standard deviation) or associated estimates of uncertainty (e.g. confidence intervals)
☐	☒	For null hypothesis testing, the test statistic (e.g. *F*, *t*, *r*) with confidence intervals, effect sizes, degrees of freedom and *P* value noted<br>*Give P values as exact values whenever suitable.*
☒	☐	For Bayesian analysis, information on the choice of priors and Markov chain Monte Carlo settings
☒	☐	For hierarchical and complex designs, identification of the appropriate level for tests and full reporting of outcomes
☐	☒	Estimates of effect sizes (e.g. Cohen's *d*, Pearson's *r*), indicating how they were calculated

Our web collection on statistics for biologists contains articles on many of the points above.

Software and code

Policy information about availability of computer code

Data collection	Metrical data were collected with MS Excel 365 for Mac (version 16.5.2) for academic use.<br>Chloride imaging data were acquired with ISee Mac Imaging software (Isee Imaging, Raleigh, NC), using their RATIOTOOL software.<br>Confocal imaging data were acquired and processed with Zen Pro and Zen Blue v3.4 software platforms, Zeiss Microscopy & Imaging (White Plains NY).
Data analysis	Data analysis of metrical data including statistical analysis was conducted with GraphPad Prism 9.1.<br>Morphometry of micrographs was conducted using Zen Blue v3.4 (Zeiss) and ImageJ (NIH-freeware).<br>Patch clamp data were analyzed using pClamp v10.3, connected to Axopatch acquisition software.<br>Human neuron image analysis was conducted using Imaris software, v9.2.1.<br>Molecular Dynamics simulation were conducted using the Desmond software, of the Desmond Molecular Dynamics System v2018.

For manuscripts utilizing custom algorithms or software that are central to the research but not yet described in published literature, software must be made available to editors/reviewers. We strongly encourage code deposition in a community repository (e.g. GitHub). See the Nature Research guidelines for submitting code & software for further information.

Data

Policy information about availability of data

All manuscripts must include a data availability statement. This statement should provide the following information, where applicable:

- Accession codes, unique identifiers, or web links for publicly available datasets
- A list of figures that have associated raw data
- A description of any restrictions on data availability

Source data file is included with the ms., data availability statement included.

Show next page (→)

Field-specific reporting

Please select the one below that is the best fit for your research. If you are not sure, read the appropriate sections before making your selection.

☒ Life sciences ☐ Behavioural & social sciences ☐ Ecological, evolutionary & environmental sciences

Life sciences study design

All studies must disclose on these points even when the disclosure is negative.

Sample size	Sample size was not calculated, but based on previous work by us (refs. PMID33731717, PMID33819485, PMID19923298), other groups and existing standards in the field. Sample sizes are given in the figure legends. Sample sizes were appropriately chosen to support conclusions.
Data exclusions	No data were excluded in this study.
Replication	Results were derived from replicated experiments, replication at least once, with equal numbers of experimental and control animals. For primary cell-based experiments, replication was conducted at least twice.
Randomization	Animals were randomized prior to experimentation. Randomization was also conducted for cell-based experiments using primary neurons as well as cultured cells.
Blinding	Investigators were blinded to treatments in animal-based and cell-based experimentation. During the primary screen, compounds were coded by numbers, and only after screening was conducted was the code un-blinded.

Reporting for specific materials, systems and methods

We require information from authors about some types of materials, experimental systems and methods used in many studies. Here, indicate whether each material, system or method listed is relevant to your study. If you are not sure if a list item applies to your research, read the appropriate section before selecting a response.

Materials & experimental systems

n/a	Involved in the study	
☐	☒	Antibodies
☐	☒	Eukaryotic cell lines
☒	☐	Palaeontology
☐	☒	Animals and other organisms
☒	☐	Human research participants
☒	☐	Clinical data

Methods

n/a	Involved in the study	
☒	☐	ChIP-seq
☒	☐	Flow cytometry
☒	☐	MRI-based neuroimaging

Antibodies

Antibodies used	We list the antibodies used in the respective table ("Supplementary Table of primary antibodies" in Suppl Mat).
Validation	https://scicrunch.org/resources<br><br>Validation<br>anti-KCC2; Millipore Cat.# 07-432; Lot : JBC1874875; RRID:AB_310611<br>anti-KCC2; Novus Cat# NBP1-74063, RRID:AB_11008395<br>anti-FLAG; SigmaAldrich Cat.# F3165; Lot# 103K6043 RRID:AB_259529<br>anti-ßIII Tubulin; Abcam (mouse) Cat.# ab78078; RRID:AB_2256751<br>anti-ßIII Tubulin; Abcam (rabbit) Cat.# ab229590; RRID:AB_2827733<br>anti-ß-catenin; SigmaAldrich Cat.# PLA0230; RRID:AB_2732045<br>anti-delta-catenin; SigmaAldrich Cat.# MABN2254; Lot Q2814922; RRID:AB_2827734<br>anti-NeuN; BioLegend Cat.# 834501; RRID:AB_2564991<br>anti-Synaptophysin; ThermoFisher Cat.# MA1-213;RRID:AB_2723681

Eukaryotic cell lines

Policy information about cell lines

Cell line source(s)	The HEK293t cell line (ATCC cat#CRL-3216) was obtained from Duke U Tissue Culture Core Facility. This cell line has been validated by ATCC (https://www.atcc.org/products/crl-3216) by short-tandem-repeat genotyping profiling. It is mycoplasma-

	free.<br>The Neuro-2a (N2a) cell line was obtained from ATCC via the Duke University Tissue Culture Core Facility (ATTC cat# CCL-131). It has not been short-tandem-repeat verified, or verified by other genomic methods, but verified for conformity with known cell morphological phenotype (https://www.atcc.org/products/ccl-131). This cell line is mycoplasma-free.
Authentication	See descriptions and comments for HEK293t and N2a cell lines.
Mycoplasma contamination	Both cell lines were tested for mycoplasma contamination.
Commonly misidentified lines (See ICLAC register)	No such cell lines were used.

Animals and other organisms

Policy information about studies involving animals; ARRIVE guidelines recommended for reporting animal research

Laboratory animals	C57BL/6J male mice (10-12 weeks old) were obtained from The Jackson Lab (Bar Harbor, ME). Kcc2-LUC mice were generated by the Liedtke Lab at Duke University and continued as a line within the Liedtke Lab mouse colony. All animal procedures were approved by The Duke University IACUC and carried out in accordance with the NIH's Guide for the Care and Use of Laboratory Animals.<br>Specifically, mice were house in a temperature and moisture controlled environment, 12/12 light-dark cycle with certified food available ad libitum as well as water.
Wild animals	Wild animals were not used.
Field-collected samples	Field-collected samples were not used.
Ethics oversight	Duke University IACUC provided animal welfare and ethics oversight.

Note that full information on the approval of the study protocol must also be provided in the manuscript.

This checklist template is licensed under a Creative Commons Attribution 4.0 International License, which permits use, sharing, adaptation, distribution and reproduction in any medium or format, as long as you give appropriate credit to the original author(s) and the source, provide a link to the Creative Commons license, and indicate if changes were made. The images or other third party material in this article are included in the article's Creative Commons license, unless indicated otherwise in a credit line to the material. If material is not included in the article's Creative Commons license and your intended use is not permitted by statutory regulation or exceeds the permitted use, you will need to obtain permission directly from the copyright holder. To view a copy of this license, visit http://creativecommons.org/licenses/by/4.0/
